# Design and Implementation of a Highly Efficient Quasi-Cyclic Low-Density Parity-Check Transceiving System Using an Overlapping Decoder

**DOI:** 10.3390/s23187828

**Published:** 2023-09-12

**Authors:** Yuxuan Sun, Liangbin Zhao, Jianguo Li, Ziyi Zhang, Xiao Yang, Xiangyuan Bu

**Affiliations:** 1 School of Information and Electronics, Beijing Institute of Technology, Beijing 100081, China; 3220210783@bit.edu.cn (Y.S.); 3120205403@bit.edu.cn (L.Z.); bxy@bit.edu.cn (X.B.); 2 School of Cyberspace Science and Technology, Beijing Institute of Technology, Beijing 100081, China; 3 Innovation Laboratory of Terahertz Biophysics, National Innovation Institute of Defense Technology, Beijing 100071, China; yx1719@126.com

**Keywords:** QC-LDPC, high-speed, 2-bit quantification, decoder, overlap, FPGA

## Abstract

The traditional LDPC encoding and decoding system is characterized by low throughput and high resource consumption, making it unsuitable for use in cost-efficient, energy-saving sensor networks. Aiming to optimize coding complexity and throughput, this paper proposes a combined design of a novel LDPC code structure and the corresponding overlapping decoding strategies. With regard to structure of LDPC code, a CCSDS-like quasi-cyclic parity check matrix (PCM) with uniform distribution of submatrices is constructed to maximize overlap depth and adapt the parallel decoding. In terms of reception decoding strategies, we use a modified 2-bit Min-Sum algorithm (MSA) that achieves a coding gain of 5 dB at a bit error rate of $10^{-6}$ compared to an uncoded BPSK, further mitigating resource consumption, and which only incurs a slight loss compared to the standard MSA. Moreover, a shift-register-based memory scheduling strategy is presented to fully utilize the quasi-cyclic characteristic and shorten the read/write latency. With proper overlap scheduling, the time consumption can be reduced by one third per iteration compared to the non-overlap algorithm. Simulation and implementation results demonstrate that our decoder can achieve a throughput up to 7.76 Gbps at a frequency of 156.25 MHz operating eight iterations, with a two-thirds resource consumption saving.

## 1. Introduction

Low-density parity-check (LDPC) codes are linear block codes that correct errors by adding parity bits, first proposed by R. Gallager in 1962 [1,2,3]. These codes are known to approach the channel capacity, with a code construction that allows them to work at a noise threshold close to the Shannon limit in memory-free symmetric channels [4,5]. In other words, LDPC codes can be transmitted at a rate lower than the channel capacity while maintaining a low bit error rate through logical coding schemes [6]. Additionally, the sparsity of the parity check matrix (PCM) used in LDPC makes it feasible to implement LDPC codes on field-programmable gate arrays (FPGAs) on a large scale [7]. As a result, LDPC codes have been widely adopted in both wired and wireless standards such as IEEE 802.3an, IEEE 802.3ba, IEEE 802.11n, IEEE 802.16e, DVB-S2, CCSDS, and others [8].

Quasi-Cyclic LDPC (QC-LDPC) codes are a type of LDPC that can offer several advantages over other error-correcting codes, including low complexity implementation, high error correction performance, flexibility, and wide usage in practical applications. They can be efficiently encoded using simple operations such as shifting and XOR [9]. Their regular structure also makes them easier to decode, and they can be designed to have a high code rate while still maintaining good error correction performance [10]. Additionally, they are also highly flexible, and can be designed to meet the requirements of different applications, including wireless communication systems, optical communication systems, and storage systems. CCSDS (8176, 7154) LDPC perfectly inherits the characteristics of QC-LDPC and maintains a high code rate, and has therefore been adopted for multiple situations [11].

The data processing speed of many specialized sensors, such as optical sensors, intelligent sensors, and biomaterial-based sensors, has been a pressing need for improvement [12,13,14]. With the intensification of the demand for communication speed in the sensor networks, the requirements for coding gain and throughput pertaining to channel coding in high-speed communication systems have correspondingly escalated [15]. While various decoding methods have been proposed, achieving high throughput with minimal hardware requirements and low power consumption remains a challenge. For instance, a LDPC decoder for 5G NR on FPGA proposed by Pourjabar achieves a max throughput of 2.2 Gbps at 10 iterations, but consumes 96 block RAMs and 225,191 look-up tables (LUT) [16]. Similarly, Sham’s decoder for a (1944, 1620) LDPC code achieves a throughput of 1.8 Gbps, requiring 100,000 registers, 65,000 LUTs, and 22 KB RAMs, which indicates low hardware utilization efficiency (HUE) [17]. And the decoder for ultra-long code with size of 149,504 × 262,144 in [18] consumes 51,000 LUTs, 1000 DSPs, and 32 Mb RAMs, reaching a throughput of 108 mbps, but can work at a very low SNR. Nevertheless, designing high-performance LDPC decoders with minimal resource requirements and low power consumption remains a formidable challenge.

The conventional decoder designs of LDPC codes, which employ the variable node processing unit (VNU) and check node processing unit (CNU) sequentially in each iteration cycle, often result in low HUE and low throughput. Although data interleaving has been proposed to enhance HUE by allowing variable node processing (VNP) and check node processing (CNP) to work on separate block data simultaneously, it doubles the memory requirements [19]. Folding is another technique that combines different block data to achieve the ideal memory depth, but sacrifices the decoder’s throughput [7]. To address these limitations, overlapped message passing has been proposed as an algorithm-level solution to overlap the CNU and VNU operations [20]. To maximize the overlap depth, which is the number of CNUs and VNUs concurrently involved in a single iteration, and enhance the throughput, a permutation vector-based LDPC code construction approach has been proposed. By using a partial parallel architecture, the proposed decoder allows column processing to commence after three row processing calculations instead of waiting for all row processing to complete, resulting in a one-third reduction in decoding time. In addition, a shift-register-based memory strategy has been employed to reduce the read/write latency.

The choice of decoding algorithm is a crucial factor affecting the performance of LDPC decoders. The Min-Sum algorithm (MSA) simplifies the multiplication operation in the decoding process to an addition operation, and approximates the summation process to finding the minimum value, thereby significantly reducing the algorithm’s complexity [21]. Building on this, the Modified 2-bit MSA optimizes the storage structure and utilizes two bits to represent the information in the operation, which further reduces the resource overhead [22].

In this paper, in response to the inability to perform deep overlap decoding for CCSDS (8176, 7154) LDPC codes, we propose a method for designing a QC-LDPC that is suitable for overlap decoding, while maintaining an equivalent submatrix structure and code length. We design a specialized partially parallel decoder based on the Modified 2-bit MSA for our proposed LDPC code, which exhibits a substantial coding gain of 5 dB at a BER of $10^{-6}$ compared to uncoded BPSK. Furthermore, when compared to the original CCSDS (8176, 7154) LDPC code with Modified 2-bit MSA decoding, our proposed LDPC maintains high performance with a marginal coding gain loss of less than 0.5 dB, while improving throughput and HUE.

This paper is organized as follows. In Section 2, QC-LDPC codes and overlapped decoding scheme are introduced, and the construction of our proposed QC-LDPC code optimized for overlap is discussed in detail. In Section 3, the Min-Sum Algorithm and Modified 2-bit MSA are reviewed. The simplification of combinatorial logic to realize the algorithm is illustrated. In Section 4, the system architecture is described, and two computation units design for decoder are presented in Gate-level netlist. In Section 5, the data stream and scheduling structure are described. The shift-register-based memory strategy and overlap controller are introduced. Section 6 analyzes the simulation results and experimental results of our decoder. Section 7 concludes this paper.

## 2. Architecture of CCSDS-like QC-LDPC Code for Overlap

### 2.1. QC-LDPC Code

QC-LDPC codes are a type of LDPC codes whose PCM can be decomposed into cyclic submatrices of equal size [23]. For an (n,k) LDPC code with a PCM of size m×n and submatrix dimension *l*, *n* and *k* represent code length before and after coding, respectively, and the PCM can be constructed using the base graph and shift factors $S_{m,n}$, where $0\leq S_{m,n}\leq l-1$. The 1s in the base graph are replaced by submatrices, and the 0s are replaced by zero matrices [24]. The origin submatrix can be either an identity matrix or a matrix derived from finite geometry, such as a double diagonal matrix. The shift factors specify the number of bits each submatrix should move to the right. For example, the cyclic submatrix I(1) is obtained by shifting the origin double diagonal submatrix one bit to the right.

$$Q\left(1\right)={\left(\begin{array}{cccccc} 0 & 1 & 1 & 0 & \cdots & 0\\ 0 & 0 & 1 & 1 & \cdots & 0\\ 0 & 0 & 0 & 1 & \cdots & 0\\ \vdots & \vdots & \vdots & \vdots & \ddots & \vdots \\ 1 & 0 & 0 & 0 & \cdots & 1\\ 1 & 1 & 0 & 0 & \cdots & 0 \end{array}\right)}_{l\times l}.$$

Specifically Q(−1) denotes the zero matrix. And in this way, we can construct H, where $m_{a},n_{b}$ indicate the number of submatrices in horizontal and vertical distribution:

$$H={\left(\begin{array}{cccc} Q(S_{1,1}) & Q(S_{1,2}) & \cdots & Q(S_{1,n_{b}})\\ Q(S_{2,1}) & Q(S_{2,2}) & \cdots & Q(S_{2,n_{b}})\\ \vdots & \vdots & \ddots & \vdots \\ Q(S_{m_{a},1}) & Q(S_{m_{a},2}) & \cdots & Q(S_{m_{a},n_{b}}) \end{array}\right)}_{m\times n},$$

**Figure 1:**
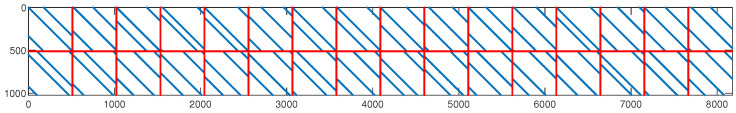
PCM of CCSDS (8176, 7154) LDPC.

The PCM of CCSDS (8176, 7154) LDPC is presented in Figure 1, whose block length is 8176 bits and the message length is 7154 bits. The PCM of CCSDS (8176, 7154) LDPC is composed of 32 submatrices in 2 rows and 16 columns. The size of submatrices is 511×511, and each submatrix satisfies the QC characteristic.

### 2.2. Overlapped Decoding Scheme

**Figure 2:**
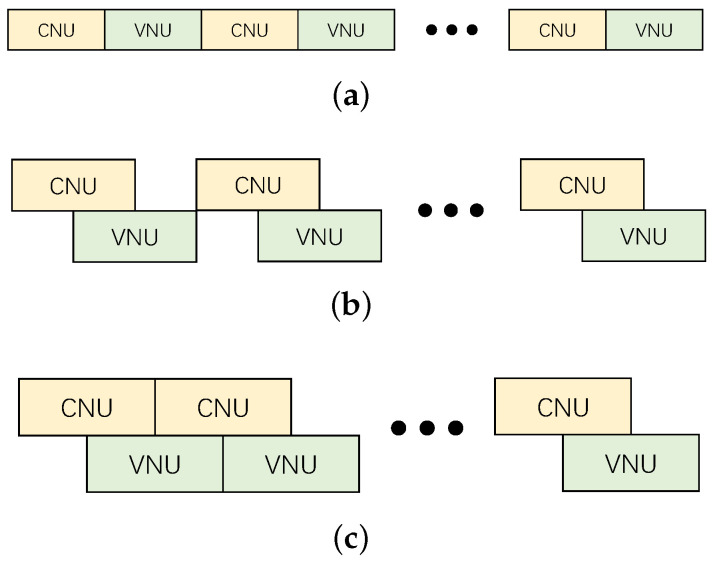
Comparison of non-overlapped, half overlapped and full overlapped decoding scheme. (**a**) non-overlapped; (**b**) half overlapped; (**c**) full overlapped.

In traditional partially parallel decoder architectures, CNU and VNU are performed sequentially in each iterative cycle, as shown in Figure 2a, resulting in low HUE and low throughput. To accelerate the decoding process, and keep low memory consumption at the same time, an overlapped decoding scheme is proposed [25]. An overlapped decoding scheme allows CNU and VNU work independently at the same time, as shown in Figure 2b, and in ideal situations, CNU and VNU can seamlessly overlap to achieve maximum throughput like Figure 2c. In this paper, we adopt an overlap scheduling structure based on the flooding decoding algorithm, which lets VNUs start iteration after a number of CNUs have been updated rather than all of them. The proposed PCM ensures that the elements required for subsequent column computations have been updated, as elaborated upon in Section 2.3. Through this approach, our decoder not only significantly enhances decoding efficiency and throughput, but also simplifies the scheduling logic and reduces logical resource consumption of the overlap controller.

### 2.3. Architecture of the Proposed QC-LDPC Code

**Figure 3:**
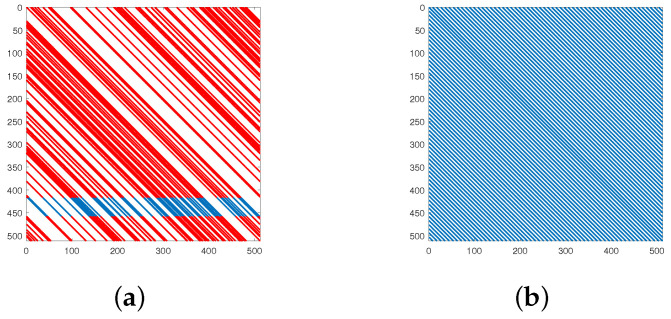
Distribution of 1s in Submatrices. (**a**) CCSDS (8176, 7154) LDPC code; (**b**) proposed (8176, 7154) LDPC code for overlap.

The indices of the first-row elements within each submatrix of the original CCSDS (8176, 7154) PCM are randomized. When arranging all the elements within submatrices into a 511×511 matrix (as shown in Figure 3a), the spacing between adjacent elements becomes randomized, while this random arrangement can yield greater coding gains, it presents challenges in applying the overlap scheduling method for further throughput enhancement during parallel decoding.

For instance, considering the original CCSDS (8176, 7154) PCM, the decoder might employ 16 VNU and 2 CNU for partial parallel iteration (where the degree of parallelism within submatrices is 1). This approach maximizes the utilization of its cyclic shift properties to save resources and increase throughput. Each VNU initiates computations in the rightwards direction from the position indexed as 1 in each column submatrix, until the current submatrix computation concludes. Therefore, while calculating VNU, it is essential that the column’s corresponding elements have been updated with the latest information from CNU iteration.

In Figure 3a, the red dots represent completed CNU iterations and updated information. If each submatrix’s VNU starts iterating from the first column, it is necessary for all submatrices’ first columns to have updated c2v messages before VNU iteration commences. Through meticulous selection, if CNU begins iterating from row 459 (cycling back to 1 after reaching 511) and reaches row 416 (the completed iteration section in red), all submatrices’ first columns will possess updated c2v messages, enabling the initiation of VNU iteration. Comparing this approach to the traditional flooding scheme, where VNU iteration must wait for CNU iteration completion, this method can let VNU start approximately 50 clock cycles earlier, before all CNU have been updated, which is also the overlap depth. Similarly, CNU can also be initiated after a certain number of VNUs have been iterated. Naturally, by selecting suitable starting points for VNU and CNU, or by using more intricate scheduling structures, the overlap depth can be extended, but introduces increased complexity in terms of hardware implementation as well.

**Figure 4:**
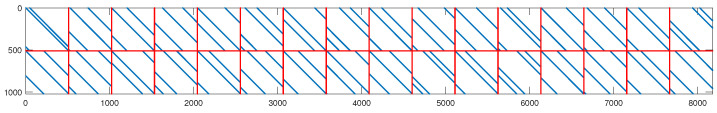
PCM of proposed (8176, 7154) LDPC codes for overlap.

Therefore, we propose a LDPC code based on CCSDS (8176, 7154) LDPC with a greater overlap depth, which is composed of 32 submatrices in 2 rows and 16 columns, and has submatrices of 511×511. In this LDPC code, the indices of first-row elements within each submatrix exhibit an approximately uniform distribution (the spacing between the last two index is smaller to fit in the 511×511 submatrix). When arranging all the elements within submatrices of this proposed PCM into a 511×511 matrix (as shown in Figure 3b, the unfolded version of which can be referred to in Figure 4), it becomes evident that the elements are nearly evenly spaced. This uniform distribution facilitates the design of more efficient and high-speed parallel overlap schemes. For the original CCSDS (8176, 7154) LDPC code, the characteristic where certain elements within specific matrices like $Q(s_{2,1})$, $Q(s_{2,3})$, ..., $Q(s_{2,15})$ have an index value of 1 is retained. For the remaining 56 elements, we introduce permutation vectors for the design process.

We employ a permutation vector shown in Algorithm 1 and defined in [26] to determine the shift factors (start index) of each submatrix, with parameters set to m=56, a=30, and b=40, where *m* stands for the number of shift factors, and a,b are parameters defined after simulations to reach a best BER performance.
**Algorithm 1:** Permutation Vector. **Input**: [m,a,b]

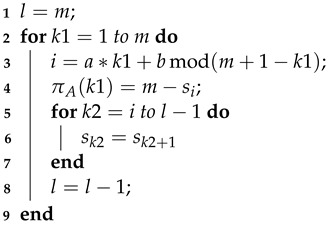
 **Output**: $\pi_{A}$


Upon obtaining the permutation vectors, we proceed to uniformly arrange the data in the submatrices with a size of 511×511, according to the shift factors from the vectors. This is achieved by multiplying the permutation vectors by ⌊511/56⌋=9 to obtain the shift factors. Moreover, to enhance the BER performance, we employ a swapping scheme that compares adjacent factors originated by the permutation vector, and exchanges them with others if their distance is too small, thus maintaining a minimum interval of two diagonals. After these steps, the PCM we constructed is displayed in Figure 4.

Utilizing the matrix we have proposed, it becomes remarkably convenient to design decoders with higher rates and lower resource consumption using the overlap scheme. When the submatrix parallelism level is set to 57, this approach can achieve convenient and regular deep overlap scheduling. The specific scheduling methodology will be discussed in subsequent sections.

## 3. Low-Complexity Decoding Algorithm

### 3.1. MSA

The process of LDPC decoding has been enhanced through the utilization of various algorithms, such as the Belief Propagation (BP) algorithm, Likelihood Ratios BP Algorithm (LLR-BP), and the MSA [27,28,29]. The MSA, in particular, has garnered widespread adoption due to its ability to simplify hardware implementation without compromising decoding performance. Notably, Fossorier contributed to further streamlining the MSA’s initialization steps by deriving formulas that enable the algorithm to decode without requiring prior knowledge of channel information [30]. In certain operations, direct assignment of initial probability values as inverses of received signal amplitudes enhances operational efficiency.

The complete MSA is shown as Algorithm 2, where rx, $it_{max}$ stand for receiving message and the default iteration time, $L(q_{n})$ stands for the soft message, N(m) stands for nodes participating in the m-th check equation, M(n) stands for check equations connected with the n-th variable node, N(m)\n stands for N(m) without the n-th node, M(n)\m stands for M(n) without the m-th equation, $r_{mn}$ is the message from the m-th check equation to the n-th node, $q_{mn}$ is the message form the i-th node to the j-th check equation, and $L\left(P\left[x_{n}|y,S\right]\right)$ is the posteriori probability.
**Algorithm 2:** Min-Sum Algorithm. **Input**: rx and PCM ***Initialization***: 
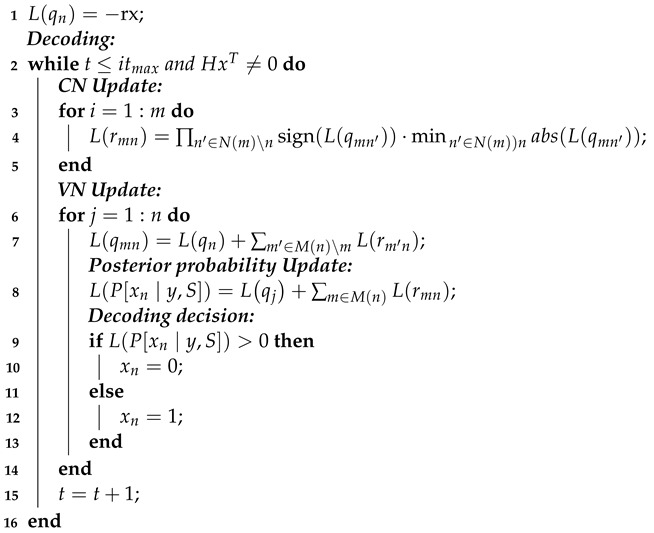
 **Output**: Decoded Data *x*

### 3.2. Modified 2-Bit MSA

In order to implement high-speed hardware for LDPC decoding, it is necessary to quantize the message values. Quantization with fewer bits can save memory and simplify the hardware structure, but it may also lead to a reduction in BER performance [31]. Based on the original MSA, [22] gave a modification only using 2-bit quantization, while retaining a good decoding performance, but greatly simplifies the computation and storage. In this algorithm, intrinsic message is quantized using 2 bits, and is represented by $B_{s}B_{m}$ [32]. Unlike traditional quantization methods, the 2-bit MSA does not directly represent the true values of the messages, but instead represents the confidence coefficient. $B_{s}$ represents a hard decision of the received message, while $B_{m}$ indicates the reliability of the hard decision [33]. When $B_{m}=0$, it represents a low confidence level but not the value of zeros, while $B_{m}=1$ indicates a high level of confidence in the hard decision. The values of $B_{s}$ and $B_{m}$ can be obtained using Equations (1) and (2), where rx is the received message and $T_{y}$ is a transformation threshold. The value of $T_{y}$ is determined as 4/10 through simulations.

(1) $$B_{s}=\mathrm{sign}\left(rx\right),$$

(2) $$B_{m}=\left\{\begin{array}{cc} 1, & \;\mathrm{if}\;|rx|>T_{y},\\ 0, & \;\mathrm{otherwise}\;. \end{array}\right.$$

After receiving the message rx, the initialization part first transforms rx from integers into 2-bit messages according to Equation (3), where $I_{n}$ stands the intrinsic message converted from rx.

(3) $$I_{n}=B_{s}B_{m}=\left\{\begin{array}{cc} 01, & \;\mathrm{if}\;rx>T_{y},\\ 00, & \;\mathrm{if}\;T_{y}\geq rx\geq 0,\\ 10, & \;\mathrm{if}\;0>rx\geq -T_{y},\\ 11, & \;\mathrm{if}\;rx<-T_{y}. \end{array}\right.$$

When implementing on FPGA, direct use of adders or comparators consumes more LUT resources compared to combinational logic circuits. To enhance the HUE and to adapt to the dual-port characteristics of the gate circuit, we have devised an improved algorithm that builds on these observations.

To optimize the memory structure, in CNU, only 3 bits are used to store each c2v message, representing the product of sign bits, min1 and min2, instead of storing all the $R_{mn}$ results. The values of min1 and min2, which are calculated in CNU, can be used to restore the exact $R_{mn}$ in VNU before use [22]. Due to the fact that the magnitude part of the CNU’s input is only 1 bit, finding min1 and min2 can be easily formulated as Boolean operations with AND and OR gates. For example, the expression for finding min1 and min2 for 8 bits is given by Equation (4).

(4) $$\begin{array}{cc} a & =B_{m1}B_{m2}B_{m3}+B_{m1}B_{m2}B_{m4}+B_{m1}B_{m3}B_{m4}+B_{m2}B_{m3}B_{m4}\\ \; & =B_{m1}B_{m2}\left(B_{m3}+B_{m4}\right)+\left(B_{m1}+B_{m2}\right)B_{m3}B_{m4},\\ b & =B_{m5}B_{m6}B_{m7}+B_{m5}B_{m6}B_{m8}+B_{m5}B_{m7}B_{m8}+B_{m6}B_{m7}B_{m8}\\ \; & =B_{m5}B_{m6}\left(B_{m7}+B_{m8}\right)+\left(B_{m5}+B_{m6}\right)B_{m7}B_{m8},\\ c & =B_{m1}B_{m2}B_{m3}B_{m4},\\ d & =B_{m5}B_{m6}B_{m7}B_{m8},\\ min_{1} & =cd,\\ min_{2} & =abc+abd+acd+bcd=ab(c+d)+(a+b)cd. \end{array}$$

To optimize the hardware implementation of the CNUs, a pipeline operation can be performed to divide the 32 inputs into four groups. This enables the calculation of four sets of min1 and min2, which can then be cascaded to a second stage by inputting these sets. By doing so, the min1 and min2 of all 32 $B_{m}$ can be obtained efficiently. This approach effectively reduces the hardware resources required for the CNU implementation, while ensuring high performance and accuracy of the decoder.

There are four steps in VNU. First, four 3-bit c2v messages are inputted based on the column weight. Next, four 2-bit $R_{mn}$ are recovered with the help of $L_{n}^{\left(k-1\right)}$, which means the $L_{n}$ in the k−1-th iteration. Then, these four $R_{mn}$ and the $I_{n}$ will be converted into integers according to Equation (6), which will be added together later. At last, the summation will be converted back to 2 bits and saved as v2c. Before going into more detail about VNU, two auxiliary functions will be introduced first. Function f(·) and g(·) are used in the VNU to convert 2-bit messages into integers for addition operation, and to transform the integer results back into 2-bit messages for storage, respectively. Specifically, f(·) is formulated in Equation (5) and g(·) is formulated in Equation (6). The magnitude bit of the integer result in f(·) corresponds to the confidence coefficient $B_{m}$, with a value of *w* for $B_{m}=0$ indicating low confidence, and a value of *W* for $B_{m}=1$ indicating high confidence. These values are determined through simulations, along with the threshold $T_{y}$ for f(·) and $T_{L}$ for the VNU process.

(5) $$f\left(B_{s}B_{m}\right)=\left\{\begin{array}{c} w,\;\mathrm{if}\;B_{s}B_{m}=00,\\ W,\;\mathrm{if}\;B_{s}B_{m}=01,\\ -w,\;\mathrm{if}\;B_{s}B_{m}=10,\\ -W,\;\mathrm{if}\;B_{s}B_{m}=11. \end{array}\right.$$

(6) $$g\left(x\right)=\left\{\begin{array}{c} 00,\;\mathrm{if}\;x\geq T_{L},\\ 01,\;\mathrm{if}\;T_{L}>x>0,\\ 10,\;\mathrm{if}\;0>x>-T_{L},\\ 11,\;\mathrm{if}\;x\leq -T_{L}. \end{array}\right.$$

And the origin computation in VNU can formulated as Equation (7) with f(·) and g(·).

(7) $$L_{mn}=L_{n}=g\left(f\left(I_{n}\right)+\sum_{j\in M(n)}f\left(R_{jn}\right)\right).$$

Ref. [34] presents a method to recover the exact $R_{mn}$ using $L_{n}^{\left(k-1\right)}$, min1, and min2, which is described in Equation (8), where $S_{m}^{\left(k\right)}$ represents the sign product of nodes connected with the m-th check equation in the k-th iteration.

(8) $$\begin{array}{cc} \left|R_{mn}^{\left(k\right)}\right| & =\min1_{m}^{\left(k\right)}+\min2_{m}^{\left(k\right)}\bar{\left|L_{n}^{\left(k-1\right)}\right|},\\ \mathrm{sign}\left(R_{mn}^{\left(k\right)}\right) & =S_{m}^{\left(k\right)}⊕\mathrm{sign}\left(L_{n}^{\left(k-1\right)}\right). \end{array}$$

Our *w* and *W* in f(·) are chosen as 1 and 5, so we need four bits to represent the four potential integers, which are −5,−1,1,5. Furthermore, during the conversion, we summarized the following connection between c2v messages’ two bits($B_{s},B_{m})$ and integers’ four bits($b_{1},b_{2},b_{3},b_{4}$) as Equation (9) shows. In this way, we are able to substitute simple logic circuit for comparers.

(9) $$\begin{array}{cc} b_{1} & =B_{s},\\ b_{2} & =B_{s}⊕B_{m},\\ b_{3} & =B_{s},\\ b_{4} & =1. \end{array}$$

The last step is the summation, which we realize using a 2-stage pipeline. At last, the first bit of the result, which is the sign bit, will be kept as $B_{s}$, and the absolute value of the result will be compared with $T_{L}$ and converted into $B_{m}$. $B_{s}$ and $B_{m}$ will make up the $L_{n}^{\left(k\right)}$, which is also the v2c messages.

## 4. Computation Unit Design for the Decoder

### 4.1. System Architecture

**Figure 5:**
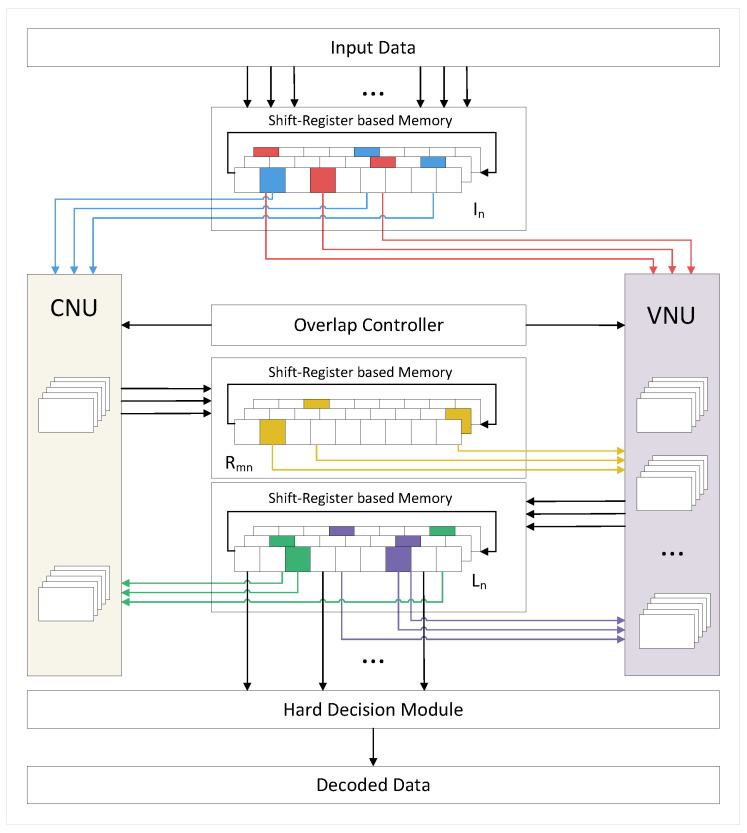
Schematic diagram of decoding system.

The architecture of our FPGA-based LDPC decoder is presented in Figure 5. It consists of several components, including CNU, VNU, shift-register-based memory, overlap controller, and hard decision module. The system utilizes a partial parallel architecture with a degree of parallelism of 2×57=114 for the CNU and 16×57=912 for the VNU.

The received soft message is quantified and truncated to 2 bits, and then stored in the shift-register based memory, which will be discussed in detail in Section 5.1. With the help of Modified 2-bit MSA, the memory efficiency is improved, and up to two thirds of memory resources can be saved.

After CNU operation, a c2v information consisting of 3 bits is generated. These 3 bits are the product of all symbol bits calculated by the XOR tree, as well as the minimum and the subminimum value of all amplitude bits. It is worth noting that in our decoder, the computation of the minimum and the subminimum values is achieved through logical operations, rather than comparators, which leads to a smaller computational delay. Furthermore, since all gate circuits are dual-ported, we further simplify the logical expression, thereby simplifying the circuit and reducing resource overhead. A detailed explanation of CNU will be provided in Section 4.2.

Once enough rows have been iterated, the overlap controller sends the synchronization signal to VNU, and the v2c result is derived after the conversion and addition. The specific operation in VNU will be introduced in Section 4.3. Both c2v and v2c messages are stored in the shift-register-based memory as well.

**Figure 6:**
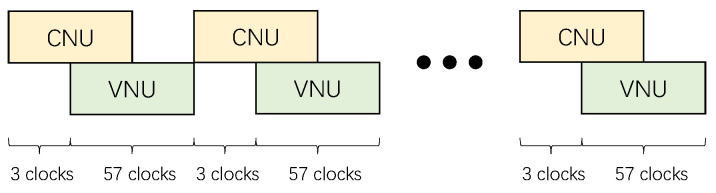
Overlap timing relationship in decoding.

Hard decisions begin when the number of iterations reaches the preset value. Using the proposed QC-LDPC matrix optimized for overlap, the overlap controller allows VNU to operate when three groups of CNU have been updated, like Figure 6 shows, and satisfied the data dependency at the same time, which accelerates decoding and triples the throughput.

### 4.2. CNU

**Figure 7:**
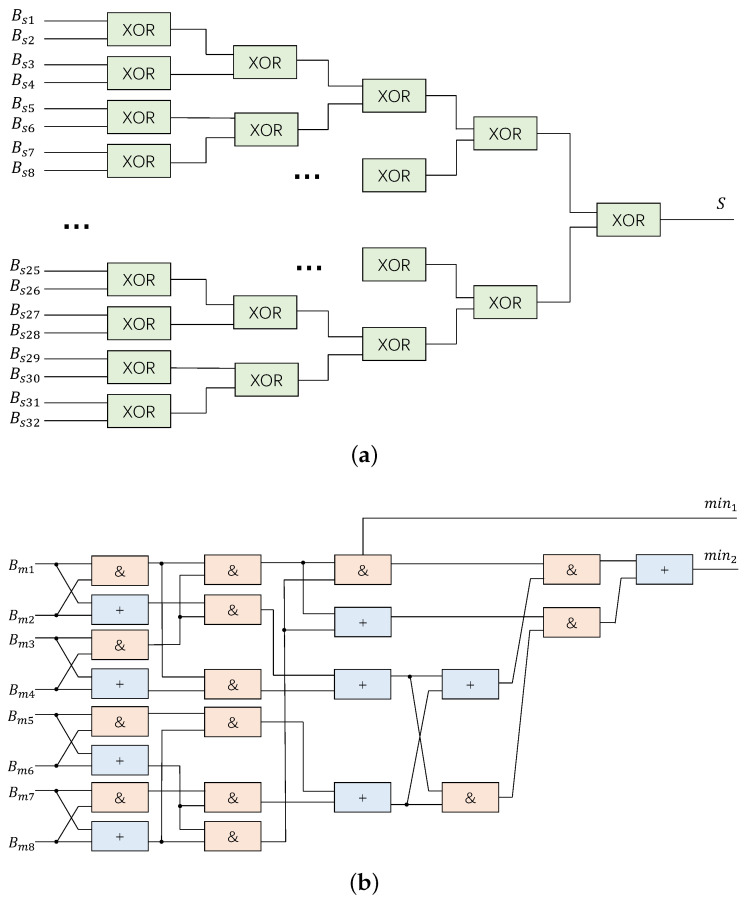
Schematic of check node unit. (**a**) XOR tree of $B_{s}$; (**b**) operation of $B_{m}$.

The schematic diagram of the CNU is presented in Figure 7. In the gate-level netlist, the XOR gate is dual-ported, which allows for a five-stage XOR tree as shown in Figure 7a. An example of the module used to determine the min1 and min2 of eight bits based on (4) is provided in Figure 7b. To obtain the min1 and min2 of all 32 bits, the output of this module is cascaded and the eight resulting values are used as inputs. The use of dual-ported characteristics simplifies the netlist, resulting in a requirement of only 239 nets and 175 cells in Vivado implementation, which is a significant reduction in resources, amounting to two-fifths of the original requirement.

### 4.3. VNU

**Figure 8:**
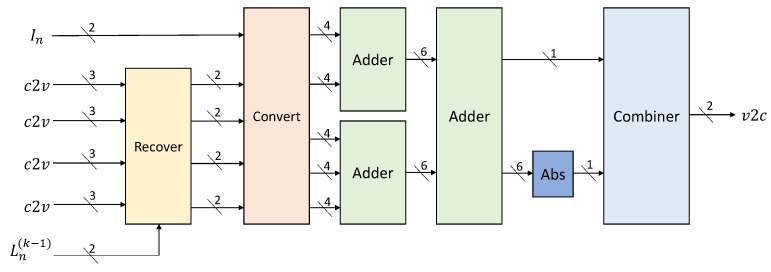
Schematic of variable node unit.

The VNU’s schematic is depicted in Figure 8, where the numbers after the slashes represent their corresponding bit widths. Firstly, four $R_{mn}$ values are generated by utilizing c2v and $L_{n}^{\left(k-1\right)}$, which will later be converted to integers. The crucial step in VNU involves adding five integers within a two-stage pipeline adder, yielding a 7-bit result that will subsequently be transformed back into 2 bits as Equation (6). The first bit is stored directly as $B_{s}$, while the remaining 6 bits are taken as absolute values based on the first bit. The absolute value is then compared to $T_{L}$, obtaining the $B_{m}$ that will be saved as v2c, along with $B_{s}$. Through a Modified 2-bit MSA and substituting partial comparators with logic circuits, the netlist is simplified, necessitating a mere 68 cells and 122 nets for each VNU in Vivado implementation.

## 5. Overlapped Decoding Scheme for the Proposed QC-LDPC Code

### 5.1. Shift-Register-Based Memory Strategy

**Figure 9:**
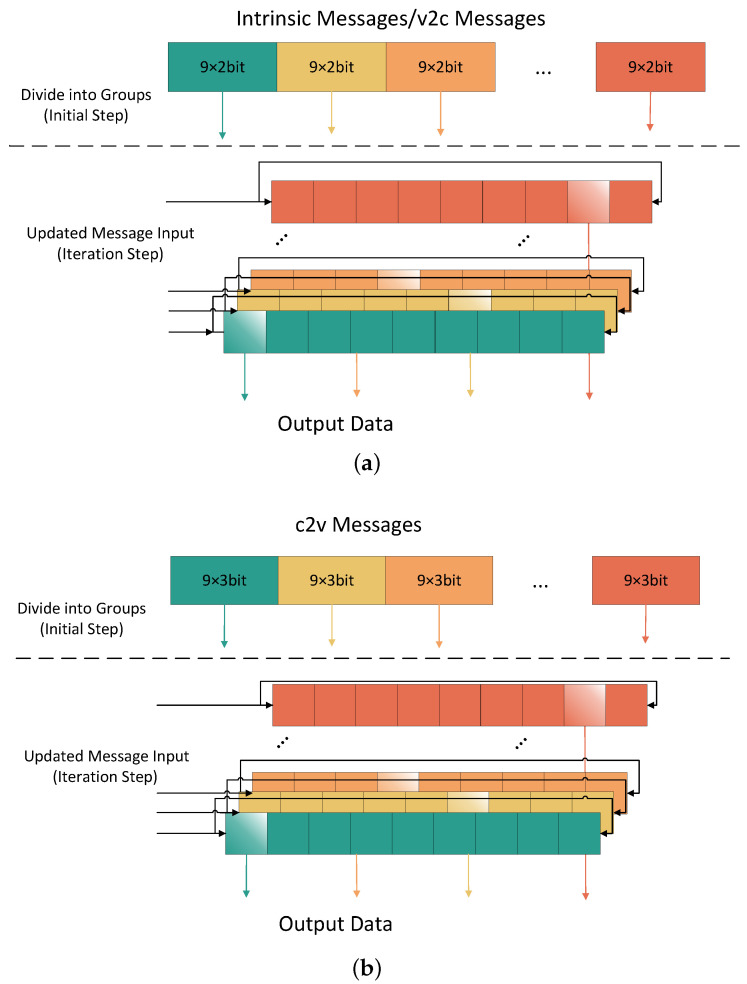
Schematic of check node unit. (**a**) Shift-register-based memory for intrinsic message and v2c message; (**b**) shift-register-based memory for c2v message.

All intrinsic messages, c2v, and v2c messages are stored in shift-register-based memory units instead of RAMs. The memory schematic we designed for overlap is depicted in Figure 9. Each row produces one c2v message per clock, and each column produces one v2c message per clock. Therefore, the data are divided into vectors and saved as an array according to the size of the submatrix in the order of rows (columns) without address conflicts.

Since the degree of parallelism is 57, we divide elements in one submatrix, dividing each of the nine columns into a group, and save in an array. Specifically, the size of registers storing the intrinsic message is [2(width)×9(length)]×57(parallelism)×16(numberofsubmatrixinarow). The size of the v2c register is 3×9×57×2, the size of the c2v register is 2×9×57×16, since each c2v message is represented by 3 bits, and each v2c message is represented by 2 bits. The shift-register-based memory unit cyclic shifts 2 or 3 bits and updates the first few bits with the latest calculation results.

In QC submatrices, every row (or column) is shifted by the previous row (or column). This means that the bits on the left of the bits processed in this clock are the bits needed for the next clock. In this way, once we decide the read port of the vector, with the cyclic shift of the register, the bits of this position are always the pending ones. This reduces the routing complexity.

Furthermore, the storage method with a fixed read/write position can eliminate the read/write latency and further accelerate the decoding process.

### 5.2. Overlap Controller

To achieve high throughput in LDPC decoding, minimizing both intra- and inter-iteration waiting times is crucial [35]. To ensure the correctness of each iteration, it is necessary to update the elements before inputting them. In the proposed PCM, which relies on the uniform distribution of diagonals, all data for the first variable node will have finished updating after the first three row processes. This allows the VNU to continue processing because the elements are saved in shift-registers.

## 6. Results and Discussion

### 6.1. Simulation Results

**Figure 10:**
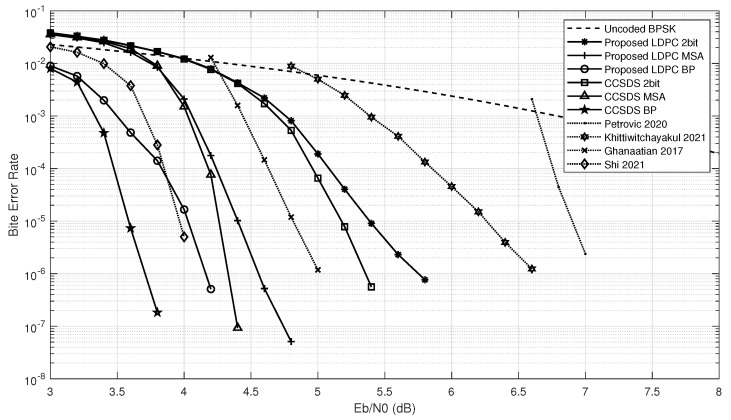
Bit error rate analysis. And performance of decoders in [36,37,38,39].

The method to determine the parameters is finding the value with best BER performance at the same SNR. After simulations in MATLAB, we determined the values of $T_{y}$, *w*, *W*, and $T_{L}$ to be 4/10, 1, 5, and 4, respectively. The BER performance simulation of proposed low-complexity high-throughput QC-LDPC decoder architecture for proposed LDPC was carried out using BPSK modulation in an AWGN channel. Figure 10 illustrates the BER performance of our implementation.

The six solid lines show the results of the MATLAB simulation. Three distinct decoding algorithms were employed, each executed over eight iterations, for both the CCSDS (8176, 7154) LDPC and the proposed LDPC code, yielding comprehensive simulation results. Initially, employing identical decoding algorithms, a minor coding gain loss of 0.5 dB was observed when applying the proposed LDPC in comparison to the original CCSDS (8176, 7154) LDPC code. This outcome underscores the effectiveness of our devised PCM, which yields a favorable performance comparable to the original CCSDS (8176, 7154) LDPC, and this 0.5 dB coding gain loss is acceptable considering that the throughput can be significantly improved by using the proposed LDPC with the same resource consumption. And, it can be found that no matter which PCM is used, there is a 1 dB coding gain loss using 2-bit MSA compared to MSA when the BER reaches $10^{-6}$. This trade-off, albeit resulting in a BER performance compromise, remains acceptable, as the 2-bit MSA significantly reduces hardware resources and complexity, all while achieving approximately two-thirds reduction in resource overhead. Overall, our decoder simultaneously increases throughput and reduces decoding overhead by sacrificing BER performance.

Meanwhile, we have selected four decoders with high throughput or low implementation complexity as the design principle and similar code rate, and compared the BER performance; the results are shown by four dashed lines in Figure 10. The decoder in [36] uses the (10368, 8448) LDPC from 5G NR with a code rate of 22/27, performs 10 iterations, employs 8-bit quantization, and the algorithm used is a layered decoding algorithm. It achieves a BER of $10^{-6}$ at a Eb/N0 greater than 7. In comparison, this decoder has higher throughput and HUE, but obviously the coding gain for the same SNR is not as good as our decoder. The decoder in [37] uses the (2048, 1723) LDPC from IEEE 802.3an with a code rate of about 7/8, five iterations, 4-bit quantization, and the decoding algorithm used is MSA. It has a SNR of 5.6 at a BER of $10^{-6}$. This decoder has extremely high throughput, and its BER performance is slightly better than ours, but its fully parallel architecture leads to significant resource overhead. The decoder in [38] uses the constructed (4608, 4096) LDPC with a code rate of 8/9, performs six iterations, uses the decoding algorithm RR-BP proposed in that article, and the encoding and decoding process are both unquantized. Its BER achieves $10^{-6}$ at a Eb/N0 of 6.7. There is no information about the implementation of this decoder, but it is clear that its BER performance lags behind our decoder by almost 2 dB. The decoder of [39] uses CCSDS (8176, 7154) LDPC with code rate 7/8, performing 20 iterations, the decoding algorithm used is NMS, and the encoding and decoding process are both unquantized. Its BER achieves $10^{-6}$ a Eb/N0 of 3.9. The NMS used by this decoder has about 0.5 dB performance improvement compared to MSA, but due to our deep overlap structure, our decoder has much higher HUE and we have higher throughput with the same resource consumption. We designed the overlap decoder using 2 bit MSA, with low resource consumption and high throughput as the design criterion. Compared to other decoders, which either have high rate but high resource consumption or low resource consumption but also low rate, our decoder can achieve a balance between resource and rate, which can meet the demand of low power consumption and high throughput well, and at the same time has a good BER performance.

**Figure 11:**
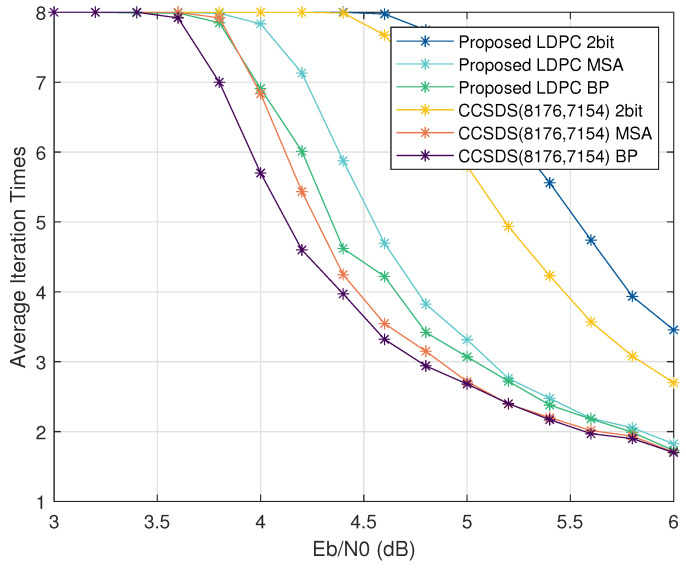
Number of iterations analysis.

As an additional facet of our analysis, Figure 11 provides insights into the average number of iterations required for successful decoding, which shows the convergence speed of the algorithms. This figure reaffirms the slight disparity introduced by our proposed LDPC code when compared to the CCSDS LDPC code at higher SNRs. Furthermore, it is evident that utilizing the 2-bit MSA leads to a performance penalty, underscoring the trade-off inherent in its application.

### 6.2. Experimental Results

For our design, we used Xilinx Virtex UltraScale+ FPGA VCU118 Evaluation Board (XCVU 9P) as the implementation platform. The throughput of a decoder is often a crucial performance metric, and it can be calculated using the following formula:

(10) $$T_{\max}=\frac{r\times l\times f}{n\times c},$$

where *r* is the code’s rate, *l* is the code’s length, *f* is the frequency of the decoder, *n* is the number of iterations, and *c* is the number of clocks per iteration [40].

For our optimized decoder designed for overlap, we can finish a single iteration in 18 clocks. Suppose we use a clock frequency of 156.25 MHz and perform eight iterations, which is a common choice for high-performance LDPC decoders. The code we use has a rate of 0.875, a length of 8176 and, thus, the throughput of the decoder can be calculated as follows:

(11) $$T_{\max}=\frac{0.875\times 8176\times 156.25\;\mathrm{MHz}}{8\times 18}=7.76\;\mathrm{Gbps}.$$

Therefore, our optimized decoder can achieve a throughput of 7.76 Gbps, which is quite high and suitable for high-speed systems. If overlap control is not used under the same decoder, one iteration takes 114 clocks, resulting in a throughput of 4.08 Gbps. In other words, overlap decoding scheme doubles throughput while maintaining unchanged resources and performance.

The utilization of the FPGA is presented in Table 1. From the table, we can find that we only need 31 LUTs per CNU and consume only 20 LUTs per VNU, which is minimal for accomplishing the comparison of 32 elements as well as the transformation and summation processing of five elements. The key to this is that we implement functions such as comparison, addition, and mapping of data through combinatorial logic, and simplify the logical relationships as much as possible. It is important to note that RAMs are not considered in this calculation, as they are not used in the shift-register-based memory scheme employed in our implementation. The permutation net, including storage, requires a total of 73,718 LUTs and 142,504 registers. Overall, our decoder utilizes only 10% of the total resources on our board. This low resource usage is a testament to the efficiency of our implementation, and demonstrates its practical feasibility for real-world applications.

Furthermore, a comparison of the implementation and performance of the proposed low-complexity high-throughput decoder with various other LDPC decoders with high HUE or similar PCM is presented in Table 2.

The findings detailed in Table 2 underscore the commendable performance of the proposed decoder in comparison to other reported decoding techniques. This achievement is particularly notable in terms of achieving a high throughput while operating at a lower working frequency. Noteworthy metrics such as Mbps/kLUT, Mbps/kFF, and Mbps/36 kb BRAM are employed for comprehensive comparison, highlighting the different HUE aspects. Our Mbps/kLUT ratio emerges as highly competitive, showcasing the efficiency of our design. However, the Mbps/kFF ratio does not distinctly favor our approach, mainly due to our utilization of shift-register-based memory as opposed to BRAMs. The high throughput is mainly achieved by relying on deep overlap with the proposed LDPC. Simultaneously, the achievement of high HUE is supported by the adoption of the 2-bit MSA and the utilization of combinational logic-based CNUs and VNUs. Evidently, the implementation results elucidate the efficacy of the design methodology proposed in this study. Its attributes of low complexity, high HUE, and impressive throughput render it a fitting solution, particularly apt for applications in sensor networks.

## 7. Conclusions

This paper presents a novel overlap-optimized decoder for high-speed communications, aimed at fulfilling the high data rate requirements of this domain. A QC-LDPC code is constructed, which allows for decoding time overlap of up to one third, thereby enhancing the decoder’s throughput. To strike a balance between decoding performance and implementation complexity, a Modified 2-bit MSA is used. In addition, a Boolean operation-based CNU and VNU is proposed to facilitate c2v/v2c message calculation by logic circuits, thereby reducing resource overhead. To address the quasi-cyclic characteristic and reduce read/write latency, a shift-register-based memory strategy is employed. The proposed decoder achieves a coding gain of 5 dB at a BER of $10^{-6}$ and attains a throughput of 7.76 Gbps at the working frequency of 156.25 MHz. Implementation results on the Xilinx Virtex UltraScale + FPGA VCU118 Evaluation Board reveal that the proposed decoder uses only 10% of the total resources of the board, attesting to its efficiency and practicality. In summary, the proposed overlap-optimized decoder provides a promising solution to meet the high data rate requirements of sensor networks.

## Figures and Tables

**Table 1:** Hardware utilization.

Computation Unit	LUT	Register	RAM	DSP
CNU	31×2×57=3534	26×2×57=2964	0	0
VNU	20×16×57=18,240	28×16×57=25,536	0	0
Permutation Net	51,944	114,004	0	0
Total	73,718	142,504	0	0
Available	1,182,240	236,4480	2160	6840

**Table 2:** Implementation and comparison of different decoders.

	Proposed Decoder	[36]	[16]	[41]	[42]
Standard	Proposed LDPC	5G NR	5G NR	CCSDS	CCSDS
Code Rate	7/8	22/27	1/3	7/8	7/8
Code Length	8176	10368	6528	8176	8176
Max. Iter.	8	5	10	10	10
Algorithm	Modified 2-bit MSA	Hybrid Schedule	OMS	F-NMS	F-AMSA
LLRs Quant.	2	8	5	7	6
Throughput (Gbps)	7.76	31.7	2.168	2	1.02
Frequency (MHz)	156.25	261	82	250	250
LUT	73,718	100,929	225,191	56,778	46,294
FFs	142,504	85,431	-	86,942	39,103
BRAM(KB)	0	4896	3456	573	253
Mbps/kLUT	106.3	314.2	9.6	35.7	22.2
Mbps/kFF	54.6	371.3	-	23	26.2
Mbps/36 kb BRAM	-	232.4	22.6	125	145
